# The association of obesity with thyroid carcinoma risk

**DOI:** 10.1002/cam4.4498

**Published:** 2022-01-15

**Authors:** Xiao‐Ni Ma, Cheng‐Xu Ma, Li‐Jie Hou, Song‐Bo Fu

**Affiliations:** ^1^ Department of Laboratory Medicine The First Hospital of Lanzhou University Lanzhou Gansu China; ^2^ The First Clinical Medical College of Lanzhou University Lanzhou Gansu China; ^3^ Department of Endocrinology The First Hospital of Lanzhou University Lanzhou Gansu China

**Keywords:** obesity, obesity‐induced TC, thyroid carcinoma, weight loss

## Abstract

**Background:**

The prevalence of obesity and an increased incidence of thyroid carcinoma (TC) threaten public health in parallel on a global scale. Sufficient evidence supports excess body fatness in thyroid carcinogenesis, and the role and anthropometric markers of obesity have been causally associated with the rising risk of TC.

**Methods:**

A literature search was conducted in PubMed. Studies focused on the effect of obesity in TC.

**Results:**

This review mainly discusses the global incidence and prevalence of obesity‐related TC. We also review the role of obesity in TC and potential clinical strategies for obesity‐related TC.

**Conclusions:**

Excess body fatness in early life and TC survival initiate adverse effects later in life.

## Abstract 2

The incidence of thyroid cancer has been increasing in the last decades all over the world. Such a steady growth cannot be entirely attributable to more sensitive diagnostic procedures. Obesity has increased with sufficient rapidity in the same time frame and may as possible promoters and modifiable risk factors for thyroid cancer in recent years. The aim of this review was to focus on the relationship between obesity and the risk of thyroid cancer. Obesity seems to play a relevant role in thyroid cancer carcinogenesis as well as in its severity and aggressiveness. The possible underlying mechanism is involved chronic inflammation mediated by cytokines, leptin, and adiponectins.

## 1 INTRODUCTION

The incidence of obesity‐related thyroid carcinoma (TC) has increased rapidly in the past few decades in different regions of the world, such as East Asia,^1^ West Asia,^2^ South Asia,^3^ Eastern Oceania,^4^ America, Europe, and the Mediterranean,^5^ particularly papillary TC, and the increasing incidence of TC may be at least partly attributed to excess body fatness.^6^ Although most TCs are generally asymptomatic and slow‐growing and patients with TC have a better life expectancy, overall mortality rates and advanced‐stage cases for TC may be a true rise.^7^ This increase in obesity‐related TC has inevitably resulted in increased global cancer rates and cost burdens. Thus, the control and prevention of obesity‐related TC have become a necessary reality.

To date, the prevalence of overweight/obesity and elevated incidence of TC have caused concern on a global scale. Current epidemiological evidence supports that obesity is a risk factor for thyroid cancer,^8^ and the prevalence of obesity has contributed to the global rise in TC incidence. Obesity was already common among the global population in 2000 and should be regarded as an epidemiological threat to global health, especially in terms of obesity‐associated cancers.^9^ Obesity‐related TC is affected by multifactorial interactions, and many studies have shown that income level, geographic influences, and genetic mutations are associated with the development of obesity‐related TC.

The prevalence of obesity and obesity‐activated TC will continue to be a concern in the coming decades, and prevention and control measures to combat obesity and obesity‐activated TC are urgently needed. In this paper, we will briefly review the relationship and the underlying mechanism between obesity and TC, as well as the prevention and control measures currently used in an attempt to reduce the incidence of obesity‐induced TC.

### 1.1 Definition of obesity categories

Anthropometric indicators of obesity are involved in body mass index (BMI), waist circumference (WC), and body fat percentage (BF%) (Table 1). BMI is the best proxy for the assessment of excess body fatness. According to obesity classification from the World Health Organization (WHO), obesity is defined as a body mass index (BMI, weight divided by square of the height) of 30 kg/m^2^ or more for white individuals and 25 kg/m^2^ or more for Asian individuals, and overweight is defined as a BMI in the range of 25–29.9 kg/m^2^ for white individuals and in the range of 23–24.9 kg/m^2^ for Asian individuals.^8^ Obesity can be further categorized into class 1, class 2, and class 3 (BMI, 30.0 to 34.9, 35.0 to 39.9, and ≥40.0, respectively). WC is the indicator of abdominal obesity (WC, ≥102 cm for men and ≥88 cm for women in White individuals, ≥90 cm for men and ≥85 cm for women in Korean adults,^10^ and ≥85 cm for men and ≥80 cm for women in the Chinese population^11^), BF% is the alternative of body fatness (BF%, males ≥ 27%; females ≥ 38%),^12^ and BMI, WC, and BF% are generally used to assess the levels of obesity. However, the definition of BMI does not consider obesity‐related morbidity and mortality or body adipose tissue distribution, and WC and BF% supply a gap in BMI.

**TABLE 1:** Definition of obesity categories

Class	BMI (kg/m^2^)		WC (cm)			BF (%)	
	White individuals	Asian individuals	Males	Females	Criterion	Males	Females
Overweight	25–29.9	23–24.9					
Obesity	≥30	≥25	≥102	≥88	WHO	≥27	38
			≥90	≥85	Korea		
			≥85	≥80	China		
Class1	30.0–34.9						
Class2	35.0–39.9						
Class3	≥40.0						

### 1.2 Global trends in the incidence of obesity and TC

Worldwide, the incidence of obesity is still increasing at an alarming rate except for the poorest areas (Table 2). The age‐standardized incidence of obesity in adults (aged 18 years or more) increased from 3.2% in 1975 to 10.8% in 2014 in men and from 6.4% to 14.9% in women.^13^ The incidence of overweight and obesity in children and adolescents in 2013 was 23.8% in boys and 22.6% in girls in developed countries and 12.9% in boys and 13.4% in girls in developing countries.^14^ An estimated 38% of the world’s adult population will be overweight, and another 20% will be obese.^15^ The overweight/obesity rate is even higher in high‐income countries/regions than in low‐ and middle‐income countries.^16^

**TABLE 2:** Global burden of obesity and TC

		Obesity			TC	
		Men	Women	Adolescents	New cases	Death cases
Incidence	2013			Boys: 23.8% Girls:22.6% or Boys:12.9% Girls:13.4%		
	2014	10.8%	14.9%			
	2016				3%–4%	
Proportion	2016	20%				
	2018				567,000	41,000

In 2018, the global burden of overall cancer was estimated to be 18.1 million new cancer cases and 9.6 million cancer deaths. Cancer statistics for adolescence and young adulthood are an estimated 1.2 million cancer cases and 400,000 cancer‐related deaths. The total estimated TC burden accounts for 567,000 new cases and 41,000 deaths cases, ranking ninth for incidence. The incidence rate of TC is three times higher in women than in men, and the estimated mortality rates are in the range of 0.4 to 0.5 in men and women.^17^ In 2016, the incidence of TC for adolescents and young adults (aged 15–39 years) increased annually by approximately 3%–4% and largely drove the overall cancer incidence in the United States.^18^ The rising incidence of TC is due to the contribution of overdiagnosis. In 2017, Bernier et al reported that there was a true increase in the incidence of TC, and they came to a similar conclusion in adults.^7^

The proportion of new cancer cases in adults worldwide attributed to a high BMI accounts for nearly 4%, the incidence of obesity‐related cancer among adolescents and young adults sharply increased from 1998 to 2012,^19^ and the prevalence of obesity seems to be a contributor to the rising incidence of TC.

### 1.3 Overview of the evidence on obesity‐related TC in humans and experimental animals

The International Agency for Research on Cancer in 2016 provided strong evidence that excess body fatness is causally associated with an increased risk of TC (see the website at https://publications.iarc.fr/570) (Table 3).

**TABLE 3:** An association of obesity with TC

Year	Types of design	Number of population	Association with thyroid cancer risk	References
2020	Retrospective investigation	457331	Positive correlation	[20]
2020	Prospective investigation	241323	Positive correlation	[21]
2020	Matched case‐control	705 TC cases and 705 controls	Positive association	[22]
2020	Matched case‐control	4977 TC cases and 19908 controls	Positive association	[23]
2019	Case‐control	10668 TC cases and 11858 controls	Positive association	[24]
2021	Prospective cohort study	3658417	Positive association	[25]
2019	Cohort study	11323006	Dose‐response association	[26]
2019	Cohort study	255051	Positive association	[27]
2020	Cohort study	9,890,917	Positive association	[1]
2018	Meta‐Analysis	17 studies	Positive association	[28]
2020	Meta‐Analysis	31 studies	Positive association	[29]
2020	Case‐control	15490 cases and 15490 controls	Positive association	[34]
2014	Cohort study	321085	Positive association	[35]
2018	Meta‐Analysis	56 studies	Positive association	[36]
2020	Cohort study	30 cases	Positive association	[37]
2017	Cohort study	783 cases	No association	[38]
2018	Cohort study	209 cases	No association	[39]

### 1.4 Epidemiologic studies in adults

Retrospective and prospective investigations showed that overweight and obesity may be important contributors to the rising incidence of PTC,^20^ and weight gain (0.4–5.0 kg/year) was positively associated with the risk of TC (HR, 1.40).^21^ A matched case‐control study showed that the adjusted ORs of the BMI categories were 1.50 for overweight and 1.62 for obesity in Korea^22^ and 1.13 for Class 1 and 1.24 for Class 2 in Korean residents.^23^ In addition, BMI and BF% were also positively associated with a significant increase in the risk of differentiated TC.^24^ A prospective cohort study in Southern Europe showed that a higher BMI was positively correlated with an increased risk of TC between 2006 and 2017, and the HR of an increase in BMI (5 kg/m^2^) was 1.08 for the risk of TC.^25^ This similar conclusion was also confirmed in the Republic of Korea^26^ in metabolically healthy (MH) and metabolically unhealthy (MUH) men. High BMI was positively associated with TC risk, whereas there was a positive relationship only in MUH women.^27^

The combined effect between obesity and metabolic syndrome was also positively associated with a higher risk of TC in men.^1^ A meta‐analysis showed that overweight and obesity were positively associated with the etiology of developing TC.^28^ Another meta‐analysis showed that obese cohorts were significantly correlated with a high risk of developing TC among women, and weight gain and annual increases in obesity indicators may be linked to the increased incidence of developing TC in both sexes.^29^

In addition, obesity seems to be more strongly associated with the prevalence of TC in women than in men, and the evidence comes from sparse studies in Korea,^30^ America,^29^ Brazil,^31^and France.^32^ Ethnicity is also considered in the epidemiological study of obesity‐related TC, although the obesity‐related increased risk of TC is generally similar between white and black men.^33^ The association of obesity with TC may be affected by sex and ethnicity.

### 1.5 Studies in childhood and adolescence

A case‐control study in the Republic of Korea provided moderate evidence that overweight and obesity in adolescence were correlated with high PTC risk in adulthood, and the association between obesity in adolescence at age 18 years and PTC risk in adulthood was stronger in men than in women.^34^ In addition to BMI, a cohort study also showed that taller height during childhood and adolescence was associated with an increased risk of adult TC.^35^ A meta‐analysis confirmed the similar conclusion that higher body fatness at a young age was positively correlated with an increased risk of developing TC later in life.^36^

### 1.6 Studies in TC survivors

Among TC survivors in a case‐control study, obesity at age 18 years was associated with cancer‐related aggressive behavior later in life, such as extrathyroidal extension and tumor size,^34^ and excess body fatness in early life will increase adverse overcomes in TC survivors. A higher BMI in TC survivors was positively associated with a higher recurrence risk of TC.^37^ However, the role of BMI in the clinical outcome of TC patients is still lack of high‐level evidence,^38^, ^39^ and further trials are warranted to determine the role of obesity in TC survivors.

### 1.7 Studies in experimental animals

In a diet‐induced obese mouse model, morphological and functional changes in thyroid glands were observed, including thyroid steatosis, distension of the endoplasmic reticulum, and mitochondrial distortion in thyroid follicular cells.^40^ In addition, the expression of lipogenesis‐regulating genes is increased, and obesity leads to morphological changes in thyroid follicular cells.^40^ To elucidate the precise association between obesity and TC, a mouse model of PTEN deficiency was fed a high‐fat diet, and obesity‐activated TC was subsequently established in a mouse model,^41^ which revealed that obesity promotes tumor growth and anaplastic changes in TC.^42^

### 1.8 The association between abdominal obesity and TC risk

In addition to BMI, WC (109 cm or more) was considered a strong predictor of TC with a sensitivity of 77.8% and specificity of 68.4% in a cross‐sectional study.^43^ WC is superior to BMI and reflects a specific index of abdominal adipose tissue distribution. Of note, current WC is positively correlated with high thyrotropin (TSH) serum levels in the general population.^44^ Since TSH acts as a sensitive indicator of thyroid function and a risk factor for TC, increasing WC may reflect the pathogenesis of TC. In a meta‐analysis, every 5 kg/m^2^ increase in BMI and 5 cm increase in WC were associated with 30% and 5% greater risks of TC, respectively, and general and abdominal obesity were positively correlated with the rising risk of TC.^45^ BMI and WC for the assessment of TC risk associated with excess body fatness showed comparable ability in a prospective cohort study.^25^

### 1.9 Effects of obesity and BMI on the histopathological features of TC

Obesity is measured by BMI, and it has been reported that obesity and BMI are associated with the clinicopathological features of TC, such as genetic mutation, NK‐cell activity, extrathyroidal extension, tumor‐node‐metastasis stage, recurrence, mortality, and pathological subtype. Evidence of the histopathological association between obesity and TC will help to establish active measures against obesity‐related TC.

1. BMI showed a positive association with the BRAF V^600E^ mutation, supporting the higher incidence of extrathyroidal extension and advanced tumor‐node‐metastasis stage in patients with papillary TC.^46^
2. Baseline BMI was more strongly associated with TC mortality and the pathological subtype of TC (papillary, follicular, and anaplastic).^45^, ^47^
3. Obesity leads to a marginally lower level of NK cell activity and helps cancer cells gobble energy immune cells.
4. Obesity increases expanded interfollicular adipose tissue or steatosis in thyroid follicular cells.^40^
5. BMI was positively correlated with TC tissue calcifications.^49^

BMI was associated with a higher risk for extrathyroidal extension and advanced tumor‐node‐metastasis stage among patients with PTC with obesity in a retrospective study.^50^ However, for the recurrence rate of TC, partial studies present inverse conclusions,^51^ and further studies will be warranted.

### 1.10 Pathophysiological mechanism between obesity and TC

The mechanisms underlying obesity‐induced TC have been proven by epidemiological studies in humans. First, increased levels of endogenous hormones have been correlated with the mechanism of TC initiation. An increase in the level of estrogen occurs, and excess estrogen is stored by adipose tissue in people with obesity and subsequently initiates TC; however, the proposed role of estrogen in TC lacks convincing human studies and may be refuted by a nationwide cohort study.^52^ Adipokines are not associated with a direct contribution to PTC,^53^ and the mechanism of adipokines in obesity‐related TC remains unclear. Insulin resistance has been confirmed by various epidemiological studies and systematic reviews to increase TC risk and facilitate cancer progression via the insulin–insulin‐like growth factor 1 axis.^54^, ^55^ Second, low‐grade chronic inflammation regulates the growth of both normal and TC cells,^56^ and elevation of human C‐reactive protein causes adult‐onset obesity.^57^ Third, leptin derived from adipocytes promotes TC cell line migration and regulates papillary TC progression.^58^ Evidence in experimental animals showed that increased leptin levels activate the JAK/STAT pathway and increase TC aggressiveness,^59^ and a case‐control study also indicated that leptin is correlated with differentiated TC.^60^ Finally, increased levels of DNA damage may participate in pathological processes, as shown in Italian adolescents with obesity.^61^

Increased systemic adiposity can contribute to steatosis in thyroid follicular cells and may result in primary hypothyroidism in patients with obesity.^40^ The expression of five obesity genes (TC FABP4, CFD, GHR, TNFRSF11B, and LTF) is significantly decreased in patients with TC, promoting TC progression and playing a role in the etiology of TC.^62^ Obesity‐associated genetic variants rs8047395 and rs8044769 were positively associated with an increased risk of TC in the matched case‐control study.^22^

Oncogenic changes in TC cells have been generally proposed to drive TC occurrence and progression. BRAF V^600E^ accounts for 60% of all TC mutations, and the prevalence of obesity is significantly related to the BRAF V^600E^ mutation in TC. This association did not change based on the presence/absence of adverse histologic features in obesity‐related TC.^63^ The link between obesity and the BRAF V^600E^ mutation in TC theoretically establishes a pathophysiological mechanism for obesity‐induced TC.^46^

In addition, increased lysyl oxidase expression in adipose tissue is positively correlated with BMI,^64^ highly expressed in aggressive TC, and associated with cancer metastasis. Patients who have the BRAF V^600E^ mutation and increased lysyl oxidase levels generally have a higher TC recurrence rate and shorter disease‐free survival time; lysyl oxidase expression is partially driven by BRAF mutation and significantly associated with the percentage of BRAF‐mutated cells, and increased lysyl oxidase levels play a key role in the higher aggressiveness of BRAF‐driven thyroid cancers.^65^

### 1.11 Effective intervention approaches to weight management

Maintaining a healthy weight is one strategy to avoid the risk of developing TC, especially in people with obesity with a family history of TC or other cancers. This strategy is the main approach to combat both obesity and obesity‐related TC before TC occurs in people with a high risk. Once TC develops, the management guidelines for these patients should take into account obesity‐related TC, and elaborately prescribed programs for weight loss should be implemented in TC survivors. Early measures to prevent obesity can contribute to avoiding or delaying the occurrence of TC in people with obesity at risk for TC, and multiple approaches can be used to prevent TC in these individuals. It would be desirable to decrease the BMI to 18.5–24.9 kg/m^2^ for White individuals and to 18.5–22.9 kg/m^2^ for Asian individuals. A BF% below 21% and 29% is recommended for men and women, respectively,^66^ and WC should be below 90/80 cm in males/females.^67^, ^68^

#### 1.11.1 Healthy lifestyle behaviors

Healthy lifestyle behaviors, which are proposed with the hope of achieving continuing education, physical activity and exercise training, and diet control, are recommended for early intervention and comprise an individual and a holistic approach for weight loss in people with obesity.^69^ The ultimate goal is to reduce the incidence of obesity‐related TC, which may at least partially be attributed to weight loss. Health education should be provided to manage people with obesity and enforce patient adherence. It is important that patients recognize the importance of the long‐term weight loss program and adhere to it to benefit from the program.

Physical activity combined with diet restriction should be an integral part of any plan to treat people with obesity regardless of weight loss goals.^70^ If possible, physical activity should be proposed to promote weight loss among people with obesity. At least 60 min/day of moderate physical activity is important for the prevention of disease and improving health in children and youth,^71^ but the intensity of physical activity can be adjusted according to the assessment of individual function in older adults with obesity. Caloric restriction in older adults with obesity remains controversial because of adverse events such as an increase in mortality. Bales notes that caloric restriction and exercise training should be combined whenever possible in older individuals with obesity.^72^ Generally, diet restriction combined with initial or delayed physical activity will lead to clinically beneficial changes in weight in a randomized trial.^73^

The success of prescribed programs for weight loss generally depends on patient adherence to generate a long‐term benefit.^74^ Personalized approaches based on changes in behavior, nutrition, and physical activity lead to more effective weight loss when obese people adhere to the regimen consistently.^75^ Diet control combined with exercise training provides a better effect on weight loss than either intervention alone, thereby shifting our clinical approach toward including behavior/lifestyle modification measures, which can contribute to cancer prevention, including thyroid cancer.

#### 1.11.2 Metformin to treat obesity‐induced TC

Drug administration is an alternative option for the treatment of obesity‐induced TC. Metformin has been investigated in preclinical and epidemiological studies, and only a few findings in humans have been reported. Metformin, the first‐line drug treatment for type 2 diabetes, has been found to prevent TC development in a Korean population in a retrospective cohort study.^76^ Similarly, metformin use has been shown to reduce the risk of TC in Taiwanese patients with type 2 diabetes.^77^ More importantly, metformin administration decreases tumor size and increases survival in patients with diabetes and TC.^78^ These findings were confirmed using a high‐fat‐diet‐induced mouse model, in which metformin blocked the invasion and metastasis of obesity‐induced TC.^79^ However, the use of metformin has not been associated with a decreased risk of TC in case‐control studies.^80^ This conclusion may be limited by the statistical power and limitations of case‐control studies. Randomized controlled trials of metformin administration against obesity‐related TC are further warranted.

#### 1.11.3 Surgical approaches for obesity‐induced TC

The mainstay of treatment in patients with TC is surgery, and obesity usually increases the difficulty of the already technically challenging surgical resection of TC and leads to delays in diagnosis. Multimodality surgery, including weight loss surgery, can contribute to weight loss and can minimize obesity and cancer issues to help accelerate recovery in patients with obesity with TC.^81^ The incidence of surgical complications in patients with obesity, such as transient hypocalcemia or transient recurrent laryngeal nerve palsy, is surprisingly high.^82^ Another cohort study showed that there is no significant association between obesity and an increase in postoperative complications, and thyroid operation can be performed safely even in patients with obesity with advanced TC.^83^

Preoperative assessment of TC aggressiveness can be performed prior to surgical planning. TC aggressiveness is positively correlated with older age, a higher BMI, a larger tumor size and the presence of the BRAF^V600E^ mutation in papillary TC.^84^ These risk predictors of extrathyroidal extension should be taken into consideration during decision making before surgery, and the surgical regimen should minimize postoperative recurrence and complications.

Postoperative follow‐up of TC surgery should be performed to monitor recurrent or residual locoregional events, and obesity has been positively associated with an increased risk of persistent or recurrent TC in patients with obesity with papillary TC (≤10 mm).^85^ These findings have been replicated and applied to postoperative recurrence surveillance in patients with obesity with TC.^86^

## 2 CONCLUSION

The prevalence of obesity increases the risk of developing TC and has been associated with the histopathological features of TC. The possible underlying mechanisms for the association of obesity with TC are involved in the changes of the level of endogenous hormones, low‐grade chronic inflammation, leptin, the level of DNA damage, steatosis and oncogenic changes in thyroid follicular cells, the expression and genetic variants of obesity genes and lysyl oxidase. We propose that receiving health education information on lifestyle changes and behavioral techniques can improve patient adherence and the execution of weight loss plans. Successful weight loss largely depends on multimodal therapy for obesity‐induced TC and may reduce the risk of developing TC.

In the future, more attention should be given to understanding the etiology and role of obesity in TC, and the evidence derived from preclinical and clinical studies should be strengthened to gain a better understanding of the mechanism underlying obesity and the occurrence of TC. Prescribing rational programs for weight loss may be essential to reduce the incidence of TC.

## CONFLICT OF INTEREST

The author(s) declare no potential conflicts of interest with respect to the research, authorship, and/or publication of this article.

## AUTHOR CONTRIBUTION

Xiao‐Ni Ma, Cheng‐Xu Ma, Li‐Jie Hou, and Song‐Bo Fu, conceived the study and wrote the paper.

## ETHICAL STATEMENT

This is a review article and the need for ethics approval and consent was waived.

## Data Availability

I confirm that I have included a citation for available data in my references section, unless my article type is exempt.
